# Accuracy of Albumin, Globulin, and Albumin–Globulin Ratio for Diagnosing Periprosthetic Joint Infection: A Systematic Review and Meta-Analysis

**DOI:** 10.3390/jcm12247512

**Published:** 2023-12-05

**Authors:** Hyonmin Choe, Emi Kamono, Koki Abe, Yuta Hieda, Hiroyuki Ike, Ken Kumagai, Naomi Kobayashi, Yutaka Inaba

**Affiliations:** 1 Department of Orthopedic Surgery, Yokohama City University, 3-9 Fukuura, Kanazawa-ku, Yokohama City 236-0004, Japan; abe.kok.sw@yokohama-cu.ac.jp (K.A.); hieda.yut.ik@yokohama-cu.ac.jp (Y.H.); hike@yokohama-cu.ac.jp (H.I.); kumagai@yokohama-cu.ac.jp (K.K.); yute0131@yokohama-cu.ac.jp (Y.I.); 2 Department of Orthopedic Surgery, Yokohama City University Medical Center, 4-57 Urahune-cho, Minami-ku, Yokohama City 232-0024, Japan; orthstud@yokohama-cu.ac.jp (E.K.); naomik58@aol.com (N.K.)

**Keywords:** periprosthetic joint infection, albumin, globulin, albumin–globulin ratio, diagnostic accuracy, systematic review, meta-analysis

## Abstract

Periprosthetic joint infection (PJI) is one of the most intractable orthopedic diseases, partly because of the difficulty in differentiating septic from aseptic conditions. We aimed to evaluate and consolidate the diagnostic accuracy of the quantitative assessment of serum albumin (Alb), globulin (Glb), and albumin–globulin ratio (AGR), alone or in combination with the inflammatory marker, C-reactive protein (CRP), for PJI. We searched the PubMed, CINAHL, and Cochrane Library databases for studies that quantitatively measured Alb, Glb, or AGR for the diagnosis of PJI up until the 30 April 2023. A total of 2339 patients were included from 10 studies, including 845 patients with a definitive diagnosis of PJI and 1494 with non-PJI. The pooled sensitivity, specificity, and area under the curve (AUC) in the summary receiver-operating characteristic curve were as follows: 0.625, 0.732, and 0.715 for Alb; 0.815, 0.857, and 0.887 for Glb; 0.753, 0.757, and 0.875 for AGR; 0.788, 0.837, and 0.876 for CRP; 0.879, 0.890, and 0.917 for the CRP–Alb ratio; and 0.845, 0.855, and 0.908 for the CRP–AGR ratio. Serum Alb, Glb, and AGR levels are feasible and accurate diagnostic markers for PJI, and the combination of these markers with CRP levels may potentially improve preoperative serum diagnostic accuracy. Future prospective studies are required to verify these findings because of the small numbers of included studies.

## 1. Introduction

Periprosthetic joint infection (PJI) is a serious complication that can occur after joint replacement surgery, such as knee or hip replacement. This refers to an infection involving tissues surrounding the artificial joint [1,2]. This infection can occur days or years following the initial joint replacement surgery. The most common bacteria associated with periprosthetic joint infections are *Staphylococcus aureus* and coagulase-negative *staphylococci*, although other types of bacteria may also be involved. Periprosthetic joint infection (PJI) is one of the most intractable diseases in the orthopedic field, partly because of the difficulty in differentiating infectious from non-infectious conditions. Diagnostic advances have contributed to improvements in the treatment of PJI [1,3,4,5,6,7,8]. Universal diagnostic criteria were established in the 2013 and 2018 International Consensus Meeting (ICM) for PJI [6,7]. The advantage of the latest 2018 ICM criteria is that the scoring system enables PJI diagnosis with a higher accuracy than before. However, one of the disadvantages of the ICM criteria is the complexity of the multifaceted diagnostic tests, which include serum biomarkers, synovial fluid tests, operative findings, and tissue examination. Some of these tests, such as microbiological cultures, histopathological assessments, and alpha defensin tests for synovial fluids, are difficult to complete preoperatively, although the diagnosis significantly affects the treatment approaches in cases of aseptic loosening or PJI.

In patients undergoing total joint replacement surgery, there is a possibility of loosening occurring between the implanted prosthesis and the bone. In such cases, revision surgery involving the removal of the loosened implant and insertion of a new implant is necessary [8]. Implant loosening can be classified as infectious (PJI) or aseptic non-infectious loosening. In non-infectious cases, replacing the implant with a new one is typically sufficient for revision surgery. However, in PJI cases, it is essential to thoroughly cleanse the bacterial focus, debride the area, and ensure bacterial eradication. In patients with PJI, performing implant revision surgery with a bacterial focus may lead to early postoperative reinfection and recurrent implant loosening. For patients with prosthetic joint infection (PJI) presenting with extensive bone loss, significant soft tissue damage, multidrug-resistant bacterial infections, or cases in which the causative pathogen is unidentified, a two-stage revision procedure is recommended [8]. This involves removing the implant to conduct infection treatment, followed by delayed re-implantation after a specified interval. As the number of surgeries increases, the damage to the bone and soft tissue also escalates, rendering the treatment of PJI challenging. Therefore, the preoperative diagnosis of infection is crucial for appropriate treatment selection and improved postoperative outcomes in patients with infectious loosening. However, diagnosing infection before revision surgery in PJI patients poses challenges, owing to the limited availability of methods. In cases of indolent infections, particularly in patients with unidentified causative pathogens, bacteria adherent to the implant periphery often form biofilms and enter a viable but non-culturable (VBNC) state [9]. This frequently results in negative bacterial cultures, making the definitive diagnosis of infection challenging even in the postoperative period. The misdiagnosis of infectious loosening as non-infectious loosening can result in patients undergoing inappropriate repeated joint replacement surgeries. To avoid the misdiagnosis of septic and aseptic loosening, universally applicable screening tests are necessary in all facilities.

Among the ICM 2018 laboratory tests, serum biomarkers are the simplest and most sensitive diagnostic methods [6]. This test has been demonstrated to be an effective screening method for PJI and should be performed as the first step in patients when there is even the slightest suspicion of PJI [1]. The ICM 2018 criteria incorporate the C-reactive protein (CRP), the erythrocyte sedimentation rate (ESR), and the D-dimer as the test parameters, and these test methods have been reported to be suitable blood diagnostic methods [6]. In contrast, culture-negative PJI reportedly shows lower CRP levels, which often complicates the detection of low-grade PJI in clinical practice [10]. Therefore, serum screening tests that can detect physical responses to infection may improve the preoperative diagnostic accuracy of PJI. In various medical fields, serum albumin (Alb) is used as a nutritional indicator and predictive marker for cancer prognosis and infectious diseases [11,12,13]. Recently, the quantitative evaluation of serum Alb and globulin (Glb) levels and the Alb–Glb ratio (AGR) have been reported to be useful in the diagnosis of PJI. This systematic review and meta-analysis aimed to evaluate and consolidate the diagnostic accuracy of serum Alb, Glb, and AGR levels, alone or in combination with the representative inflammatory marker, CRP, for PJI.

## 2. Materials and Methods

This systematic review and meta-analysis was performed in accordance with the Preferred Reporting Items for Systematic Reviews and Meta-Analyses (PRISMA) guidelines [14]. Institutional Review Board approval was not required for this study.

Two researchers (HC and KA) systematically conducted electronic searches to identify all eligible articles in the PubMed, CINAHL, and Cochrane Library databases. The following search terms were used: “prosthesis related infections”[Mesh Terms; MH] OR “prosthesis related infections”[Title/Abstract; TIAB] OR “prosthesis related infection”[TIAB] OR “prosthesis related infection”[TIAB] OR “Periprosthetic joint infection”[TIAB]) AND (“albumins”[MH] OR “albumin*”[TIAB] OR (“globulins”[MH] OR “globulin*”[TIAB])) AND (“Sensitivity and Specificity”[MH] OR “diagnosis accuracy”[TIAB] OR “sensitivity”[TIAB] OR “specificity”[TIAB] OR “diagnostic accuracy”[TIAB]”. The literature search was performed until the 30 April 2023.

### 2.1. Study Screening and Eligibility Assessment

Two reviewers (HC and KA) screened the titles and abstracts. After the removal of duplicates and exclusion by abstract and title screening, further studies were performed for full-text article assessments. Studies were included if they met the following criteria: (1) Alb, Glb, and AGR levels were quantitatively measured as serum biomarkers for the diagnosis of PJI; (2) the ICM 2013 or 2018 criteria were used for the definitive diagnosis of PJI; and (3) the accuracy of the serum biomarkers was determined directly or indirectly.

### 2.2. Data Extraction and Quality Assessment

The relevant data were independently extracted by two researchers (HC and KA). The Quality Assessment of Diagnostic Accuracy Studies 2 (QUADAS-2) tool was used to evaluate the quality of the literature. The QUADAS-2 tool is a methodological tool used for the quality assessment of diagnostic accuracy studies. This tool was developed to address limitations of the original QUADAS tool and provide a structured framework for evaluating the risk of bias and applicability of primary diagnostic accuracy studies. The QUADAS-2 tool includes patient selection, an index test, a reference standard, and flow and timing [15]. If there were any disagreements during this process, a third author was responsible for making the decision.

### 2.3. Statistical Analysis

A two-by-two table containing the number of true-positive, true-negative, false-positive, and false-negative results was constructed for each study. Then, the two-by-two data was entered into Review Manager 5 (Review Manager 2014). Estimates of sensitivity and specificity were summarized for each individual study using forest plots. We used a funnel plot for assessing publication bias and visually assessing asymmetry. Egger’s test was employed for index tests with ten or more adopted studies.

A bivariate random-effect meta-analysis that generates a summary receiver-operated curve (SROC) with the calculated area under the curve was conducted. A summary estimate of the sensitivity and specificity, with confidence intervals (CI) creating a 95% confidence region ellipse on the SROC, was also calculated. All the meta-analyses were performed using the mada package in R version 4.3.1. The pooled estimates of sensitivity and specificity were plotted using SROC plots in Review Manager 5 (Review Manager 2014). Heterogeneity was assessed visually using both forest plots and SROC plots. The quality of evidence was judged using the GRADE framework.

## 3. Results

**Figure 1:**
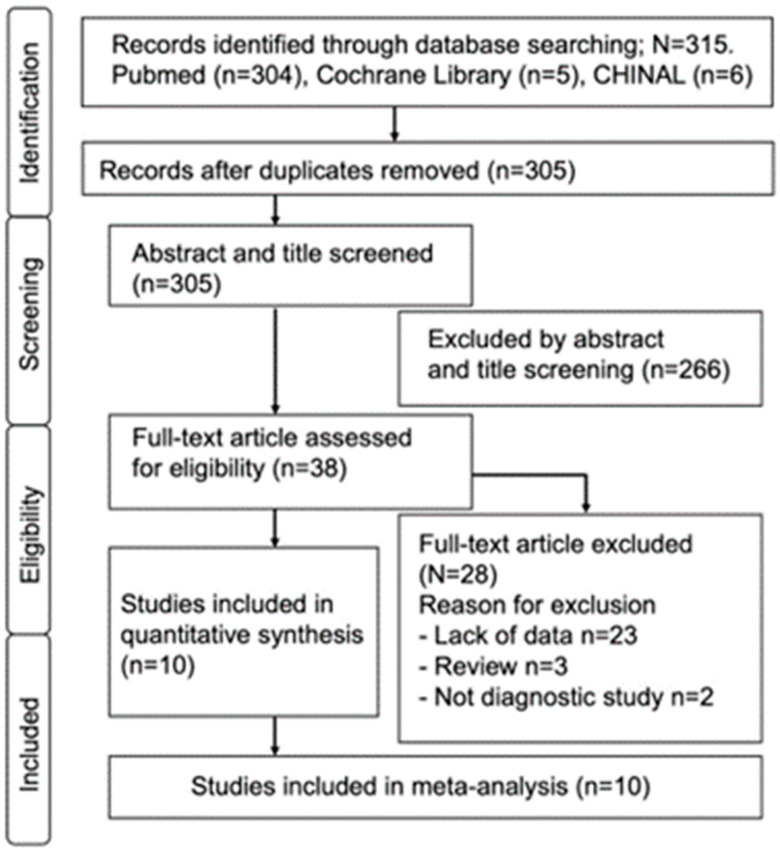
Flow diagram of the study selection process.

A flowchart of the study screening and eligibility process is shown in Figure 1. First, a database search identified 315 articles. The removal of duplicates and exclusion of inappropriate studies yielded 38 studies, of which 266 were excluded by abstract and title evaluation. Thirty-eight studies were subjected to full-test scrutiny, resulting in the exclusion of an additional 28 studies. As a result, 10 studies were deemed eligible for the meta-analysis (Figure 1) [16,17,18,19,20,21,22,23,24,25]. A total of 2339 patients were enrolled from these 10 studies, including 845 patients with a definitive diagnosis of PJI and 1494 patients without PJI. Six studies applied the ICM 2013 criteria [7] as the definitive diagnostic criteria for PJI, while the other studies applied the 2018 ICM criteria [6] (Table 1). All the studies were retrospective and included only patients who underwent knee or hip joint total hip arthroplasty.

### 3.1. Quality Assessment and Risk of Bias

**Figure 2:**
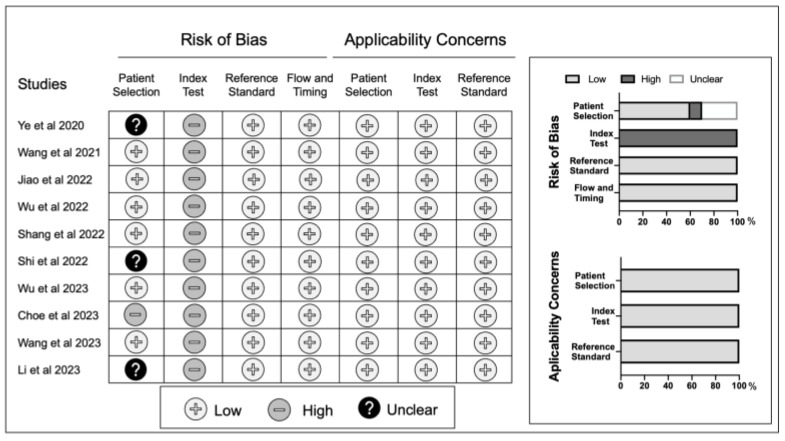
Quality assessment of the included studies based on the Quality Assessment of Diagnostic Accuracy Studies 2 tool criteria [16,17,18,19,20,21,22,23,24,25].

The quality assessment results of the QUADAS-2 scales for the 10 studies are shown in Figure 2. The results indicated that the overall quality of the included studies was mostly “low risk”, except for the risk of bias in the index test. The reason for this “high risk” result was because all the studies retrospectively analyzed diagnostic accuracy using Youden’s Index [26]. The “unclear” risk of bias results for patient selection in three studies were because these studies lacked a description for the patients with inconclusive cases, as per the ICM 2018 PJI definition. The “high” risk of bias results for patient selection in one study was because only this study included osteoarthritis patients. Although the sample sizes of the included studies were relatively small, the quality of their research was persuasive. In the funnel plot, visual asymmetry was noted in the index tests, excluding AGR. Since the CRP test was the only index that included more than 10 studies, Egger’s test was specifically conducted for the CRP test. The significant result of Egger’s test (*p* = 0.0453) suggests the existence of publication bias in the CRP test.

### 3.2. Meta-Analysis of Diagnostic Accuracy for PJI

**Figure 3:**
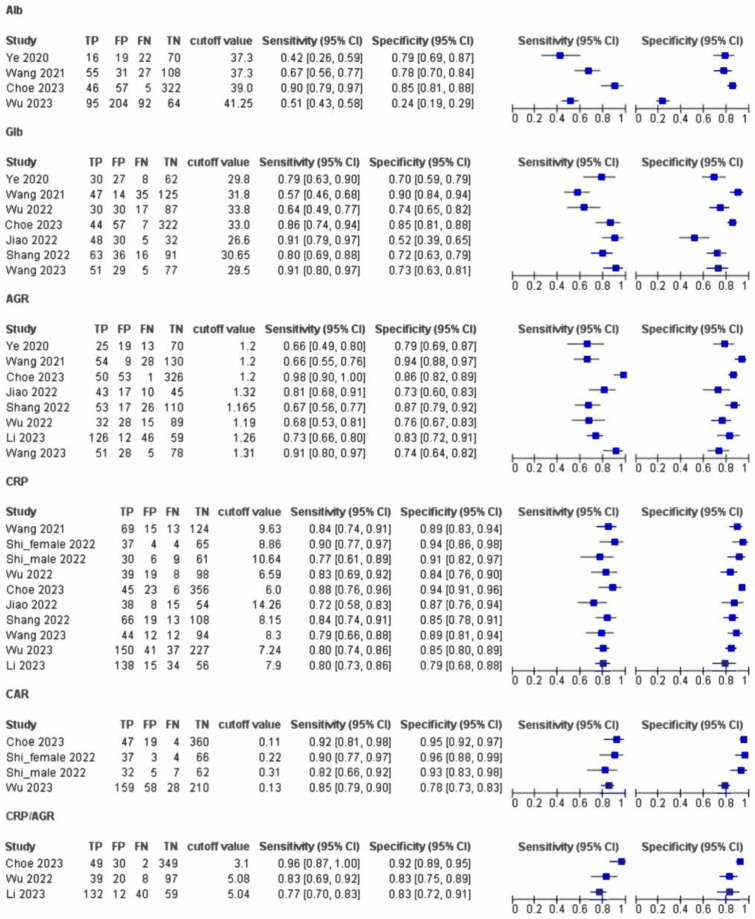
Forest plot of sensitivity and specificity for the serum marker. AGR, albumin to globulin ratio [16,17,18,19,20,21,22,23,24,25]; ALB, albumin; CAR, CRP–albumin ratio; CI, confidence interval; CRP, C-Reactive protein; FN, false-negative; FP, false-positive; GLB, globulin; TN, true-negative; TP, true-positive.

**Figure 4:**
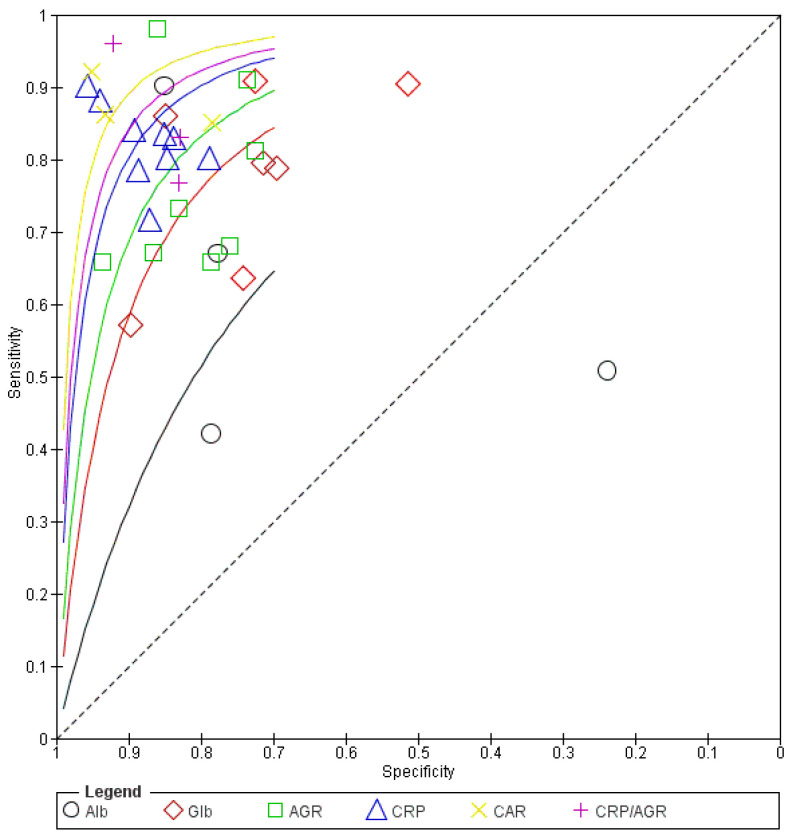
Summary receiver-operating characteristic curve of the included studies. AGR, albumin–globulin ratio; Alb, albumin; CAR, CRP–albumin ratio; CRP, C-reactive protein; Glb, globulin.

The pooled sensitivity and specificity were the following: 0.625 (95% confidence interval [CI]: 0.374–0.823) and 0.732 (95% CI: 0.385–0.922) for Alb; 0.815 (95% CI: 0.761–0.859) and 0.857 (95% CI: 0.801–0.900) for Glb; 0.753 (95% CI: 0.570–0.875) and 0.757 (95% CI: 0.581–0.875) for AGR; and 0.788 (95% CI: 0.726–0.839) and 0.837 (95% CI: 0.778–0.883) for CRP (Table 2). The combination markers of the CRP–Alb ratio (CAR) and CRP–AGR ratio (CAGR) improved the diagnostic accuracy of these markers, with pooled sensitivities of 0.879 (95% CI: 0.830–0.916) and 0.845 (0.704–0.926) and specificities of 0.890 (95% CI: 0.782–0.948) and 0.855 (0.633–0.953) (Table 2, Figure 3). The cutoff values established in each study widely varied from study to study for Alb (37 to 42 g/L), Glb (26 to 32 g/L), CRP (2.0 to 14.3 mg/L), and combination markers of CAR (0.11–0.22) and CRP/AGR (3.1–5.1), except for AGR, which had consistent cutoff values between 1.165 and 1.32 among eight papers. The SROC curve demonstrated that the AUC for Alb, Glb, AGR, CRP, CAR, and CRP/AGR were 0.715, 0.887, 0.819, 0.876, 0.917, and 0.908, respectively (Figure 4). The forest plots (Figure 3) and SROC curves (Figure 3) reveal variations in sensitivity and specificity across all the index tests, indicating heterogeneity. The quality of evidence of the Alb test for diagnosing PJI was low because the estimates vary widely for both sensitivity and specificity, with a wide confidence interval, and that of the Glb was moderate because the observed data were far from the SROC curve and varied along the curve. The quality of evidence of the CRP test was moderate and publication bias was strongly suspected. The quality of evidence of the AGR test was low because the observed data were far from the SROC curve and varied along the curve, with a wide confidence interval.

## 4. Discussion

Diagnosis before surgery is essential for the appropriate treatment of PJI, as there are significantly different treatment approaches for aseptic loosening. In the field of orthopedic infectious diseases, diagnostic imaging using computed tomography (CT) or Magnetic Resonance Imaging (MRI) is commonly employed for the identification of infectious regions in the bone and soft tissue. However, in cases of prosthetic joint infection (PJI), the presence of artifacts due to implants makes diagnostic imaging using CT and MRI challenging. Instead, nuclear medicine modalities have been reported to be useful in the diagnosis of PJI [1,27,28,29,30]. Nevertheless, nuclear medicine exams pose challenges in terms of cost and facility requirements as standard imaging diagnostics, making their use in routine preoperative screening difficult. Therefore, for the preoperative diagnosis of PJI, blood tests and joint synovial fluid analyses are essential. Furthermore, even when synovial fluid aspiration is performed before surgery, patients from whom joint fluid cannot be collected due to a dry tap necessitate screening solely through blood tests. The 2013 and 2018 ICM criteria are the globally recognized diagnostic criteria for PJI [6,7]. These multifaceted diagnostic criteria have improved the accuracy of PJI diagnosis. However, these criteria include diagnostic methods that are difficult to implement in general hospitals and some diagnostic tests that yield results only after surgery. Hence, a simple screening test that can be completed before surgery, is feasible in general hospitals, and has sufficient accuracy is in great demand for patients with PJI, especially in patients presenting with a dry tap for synovial fluid and difficulty in preoperatively achieving a definitive diagnosis.

Among various infectious diseases, preoperative serum tests are the most convenient and effective method of preoperative screening. The C-reactive protein (CRP) test is the most straightforward and convenient method for quantitatively assessing inflammation and is commonly used to evaluate the intensity of infection and inflammation in various infectious diseases. The CRP, discovered in 1930 by William Smith Tillett and Thomas Francis in the United States, undergoes precipitation reactions with the C-polysaccharide of pneumococci [31,32]. It activates the immune response against bacteria by binding to them, and its elevation of production during bacterial infections has led to its widespread use in clinical settings for the diagnosis and assessment of infections [31,32]. Its high accuracy has led to its recommendation for screening in the diagnosis of prosthetic joint infection (PJI) [1,6]. The erythrocyte sedimentation rate (ESR) test measures the rate at which red blood cells settle in a solution. The causes of an elevated ESR include a decrease in red blood cells or albumin levels and an increase in gamma globulin or fibrinogen levels.

The combination of CRP and ESR is recommended in the diagnostic flowchart for PJI proposed by the American Academy of Orthopaedic Surgeons (AAOS) as an early and simple screening method [1,33]. Indeed, the combination of CRP and ESR has been widely reported to maintain excellent accuracy in PJI diagnosis. In the 2018 International Consensus Meeting (ICM), the measurement of the D-dimer was also recommended [6]. The D-dimer, a fibrin degradation product yielded when fibrin is broken down by plasmin, has been a crucial test in blood examinations for suspected thrombotic disorders, such as venous thromboembolism, since its introduction in the 1990s [31,32]. Although the D-dimer has been reported as a useful serological diagnostic method for PJI, conflicting reports on its utility in PJI diagnosis exist [34]. Numerous reports have suggested the usefulness of other coagulation and fibrinolysis system blood test markers in PJI diagnosis [35]. In recent years, various blood markers have been applied in the diagnosis of PJI, including ratios such as the neutrophil-to-lymphocyte ratio and the platelet count, which have also been reported as useful indicators of a patient’s immune status in the diagnosis of PJI [35].

Among diverse patient populations, however, there are individuals who exhibit low levels of inflammatory markers, even in the presence of bacterial infections, due to comorbidities and concurrent treatment, or patients who exhibit high CRP levels even in the absence of bacterial infections [10,36]. Recently, a combination of various blood markers has been reported to be useful for improving the accuracy of infectious disease diagnosis compared to a single inflammation marker [35,37]. Among serum markers, Alb and Glb are widely used in the diagnosis or prediction of malignant tumors, postoperative mortality, and orthopedic infectious diseases [21,38,39,40,41,42]. Therefore, our systematic review and meta-analysis evaluated the usefulness of the serum immune markers Alb and Glb and the combination index of these markers.

Albumin is a protein predominantly synthesized in the liver and serves as a straightforward indicator of nutritional status through simple blood tests [43]. In patients undergoing orthopedic surgery, low levels of albumin are recognized as a significant risk factor that independently induces various complications [12]. Following the onset of infection, trauma, and inflammation, storage proteins in the body such as muscles and albumin undergo degradation, leading to the enhanced production of the inflammatory protein CRP (C-reactive protein) to activate the immune response [43]. Alb is widely associated with the immune system and performs various biological functions [43]. Low albumin levels suggest decreased immune response and malnutrition. Moreover, elevated levels of CRP in conjunction with low albumin levels indicate a decline in albumin production and increased breakdown due to inflammation or infection. Alb levels are negatively correlated with CRP levels owing to the biological effect of interleukin (IL)-6 on hepatocytes, which increases CRP production and reduces Alb production [44]. Glb, also known as immunoglobulin, can be quantified by subtracting Alb from the total protein in serum examinations [43], and, in patients with infectious conditions, IL-6 production also increases immunoglobulin levels. Thus, Glb levels are representative of antibody production and have been used to evaluate the overall status of immune responses to infections. As the sum of Alb and Glb is equal to the total protein level, the AGR sensitively reflects the immune activation of individuals, and this biological pathway may explain the high accuracy of Glb, AGR, CAR, and CRP/AGR tests in our meta-analysis. Interestingly, the optimal cutoff value for AGR screening was 1.1 or 1.2 in four different studies, indicating the consistency of these serum tests, although the other biomarkers showed widely variable cutoff values among the studies. The choice of cutoff values significantly affects both sensitivity and specificity; however, the optimal cutoff values for diagnostic markers can differ based on the patient background or bacterial organisms [10,24]. Therefore, the AGR can be considered a reliable diagnostic tool because it showed consistent cutoff values in many studies.

Serum Alb, Glb, and AGR measurement is a commonly used blood screening method for nutrients and inflammatory disorders in general hospitals, alongside the serum CRP measurement and combination indices of AGR, CAR, and CAGR [23]. In our study, these combination indices were demonstrated to have a high sensitivity and may possibly provide a better diagnosis without additional invasive procedures or new technology. The cumulative AUC for the AGR was favorable (0.84). Moreover, the AUC for the combination marker of CRP/AGR was 0.91, which was higher than the diagnostic accuracy of CRP alone. This suggests that the AGR can identify PJI cases with abnormal values, which may be difficult to screen using CRP alone. Indeed, two studies demonstrated the utility of the AGR in the detection of low-grade PJI, defined as PJI with <10 mg/L [21,25]. This implies that, by incorporating immunological markers, such as Alb and Glb, in addition to inflammatory markers, such as CRP, blood-based PJI screening can be performed with a higher sensitivity. Many tests offer the flexibility to set different cutoff values, allowing them to be employed as highly sensitive screening tools or as more specific diagnostic tests, depending on their biological characteristics. Thus, it may be possible to improve the preoperative diagnostic accuracy of PJI by setting two different cutoff values for each serum biomarker and assigning different scores in the current scoring system for PJI diagnosis. If the two highly specific markers were elevated to a significant degree, it would enable the preoperative diagnosis of PJI using only serum tests. This serum diagnosis could improve the accuracy of the preoperative diagnosis of PJI and facilitate a treatment approach focused on addressing infection. For this purpose, it is essential to combine two distinct serum markers with different biological characteristics to eliminate potential patient-related factors for misleading diagnoses. Alb, Glb, and AGR are effective potential candidates for the diagnosis of PJI.

This study had several limitations. This systematic review and meta-analysis study included a small number of retrospective diagnostic studies owing to the rarity of PJI and aseptic loosening and the novelty of the application of Alb and Glb in PJI. An overestimation of test results and a lack of generalizability may be present, highlighting the need for further prospective validation with a larger patient population in the future. Another limitation worth mentioning is that there was a lack of documentation regarding the handling of inconclusive PJI cases in many of the studies utilizing the 2018 ICM criteria, although the diagnosis of inconclusive cases often poses a significant challenge in clinical practice. In the future, it will be necessary to further investigate the utility of these immunological markers in stratified analyses of inconclusive cases or low-grade PJI. While these limitations pose challenges for future research, this systematic review and meta-analysis indicated the potential usefulness of Alb, Glb, and AGR, in combination with CRP (CAR or CRP/AGR), as feasible and accurate blood screening tools for PJI.

In conclusion, serum Alb, Glb, and AGR levels in combination with CRP levels are feasible and accurate diagnostic markers for PJI and can improve the preoperative diagnostic accuracy of PJI.

## Figures and Tables

**Table 1:** Characteristics of the studies included.

Study	Year	Study Design	Number of Patients	Number of PJI	ICM Criteria	Serum Biomarkers
Ye et al. [16]	2020	R	127	38	2018	Alb, Glb, AGR
Wang et al. [17]	2021	R	221	82	2013	Alb, Glb, AGR, CRP
Jiao et al. [18]	2022	R	115	53	2013	Glb, AGR, CRP
Wu et al. [19]	2022	R	164	47	2013	Glb, AGR, CRP, CRP/AGR
Shang et al. [20]	2022	R	206	79	2013	Glb, AGR, CRP
Shi et al. [24]	2022	R	216	80	2018	CRP, CAR
Wu et al. [23]	2023	R	445	187	2013	Alb, CRP, CAR
Choe et al. [21]	2023	R	430	51	2018	Alb, Glb, AGR, CRP, CAR, CRP/AGR
Wang et al. [22]	2023	R	162	56	2013	Glb, AGR, CRP
Li et al. [25]	2023	R	243	172	2018	AGR, CRP, CRP/AGR

R, retrospective study; AGR, albumin–globulin ratio; Alb, albumin; CAR, CRP–albumin ratio; CRP, C-reactive protein; Glb, globulin; ICM, International Consensus Meeting; PJI, periprosthetic joint infection.

**Table 2:** Pooled sensitivity and specificity and area under the curve.

	Sensitivity			Specificity			Area under the Curve
Index	Estimate	95%ci.lb	95%ci.ub	Estimate	95%ci.lb	95%ci.ub	
Alb (n = 4)	0.625	0.374	0.823	0.732	0.385	0.922	0.715
Glb (n = 7)	0.815	0.761	0.859	0.857	0.801	0.900	0.887
AGR (n = 8)	0.753	0.570	0.875	0.757	0.581	0.875	0.819
CRP(n = 9)	0.788	0.726	0.839	0.837	0.778	0.883	0.876
CAR (n = 3)	0.879	0.830	0.916	0.890	0.782	0.948	0.917
CRP/AGR (n = 3)	0.845	0.704	0.926	0.855	0.633	0.953	0.908

AGR, albumin–globulin ratio; Alb, albumin; ci.lb, confidence interval lower-bound; ci.ub, confidence interval upper-bound; CAR, CRP–albumin ratio; CRP, C-reactive protein; Glb, globulin.

## Data Availability

The datasets used and/or analyzed during the current study are available from the corresponding author on reasonable request.
